# Supplementary figures and images for: Oral zinc sulphate reduces the recurrence rate and provides significant therapeutic effects for viral warts: A systematic review and meta-analysis of randomized controlled trials
Source: PLoS One. 2025 May 7;20(5):e0323051. doi: 10.1371/journal.pone.0323051 (PMC12058182; doi:10.1371/journal.pone.0323051)

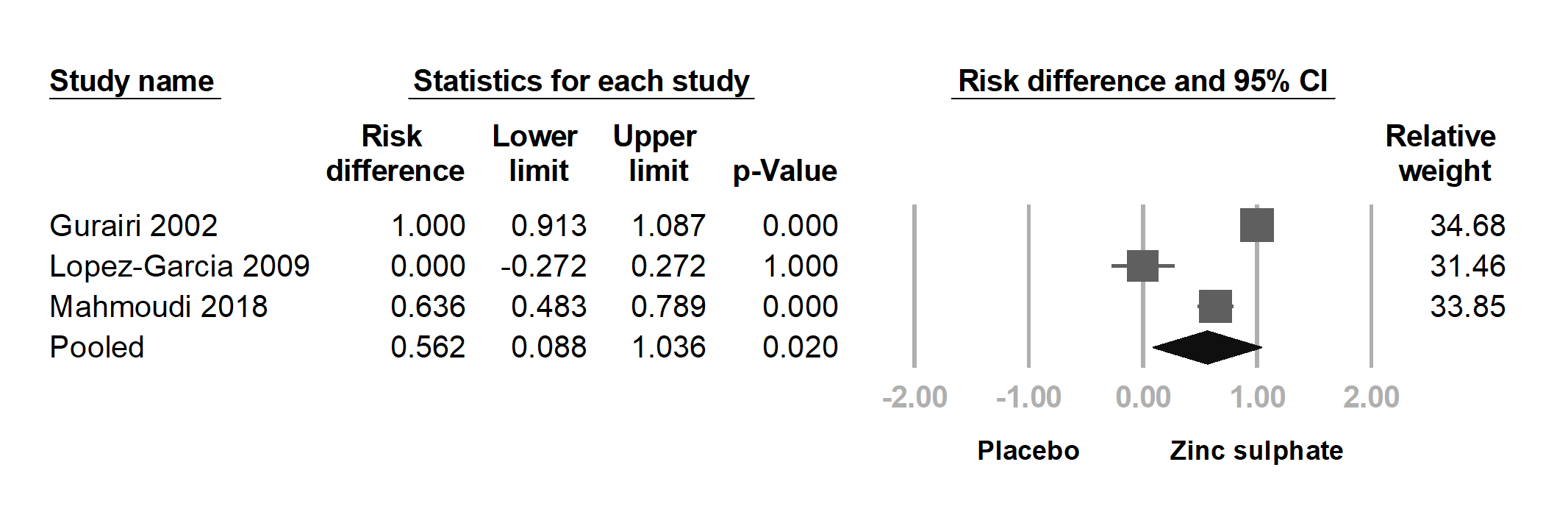

Supplement: S1 Fig — Nausea was significantly more frequent in patients taking zinc sulphate. The studies are listed in alphabetical order. CI, confidence interval. (TIF) [file pone.0323051.s001.tif]

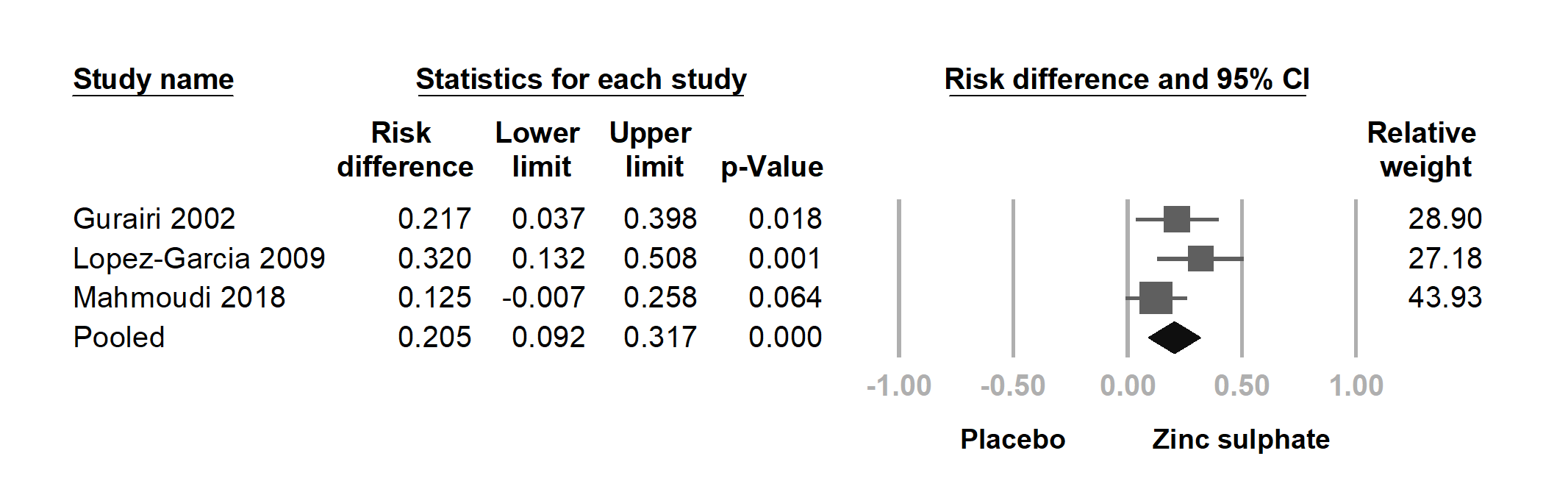

Supplement: S2 Fig — Vomiting was significantly more frequent in patients taking zinc sulphate. The studies are listed in alphabetical order. CI, confidence interval. (TIF) [file pone.0323051.s002.tif]

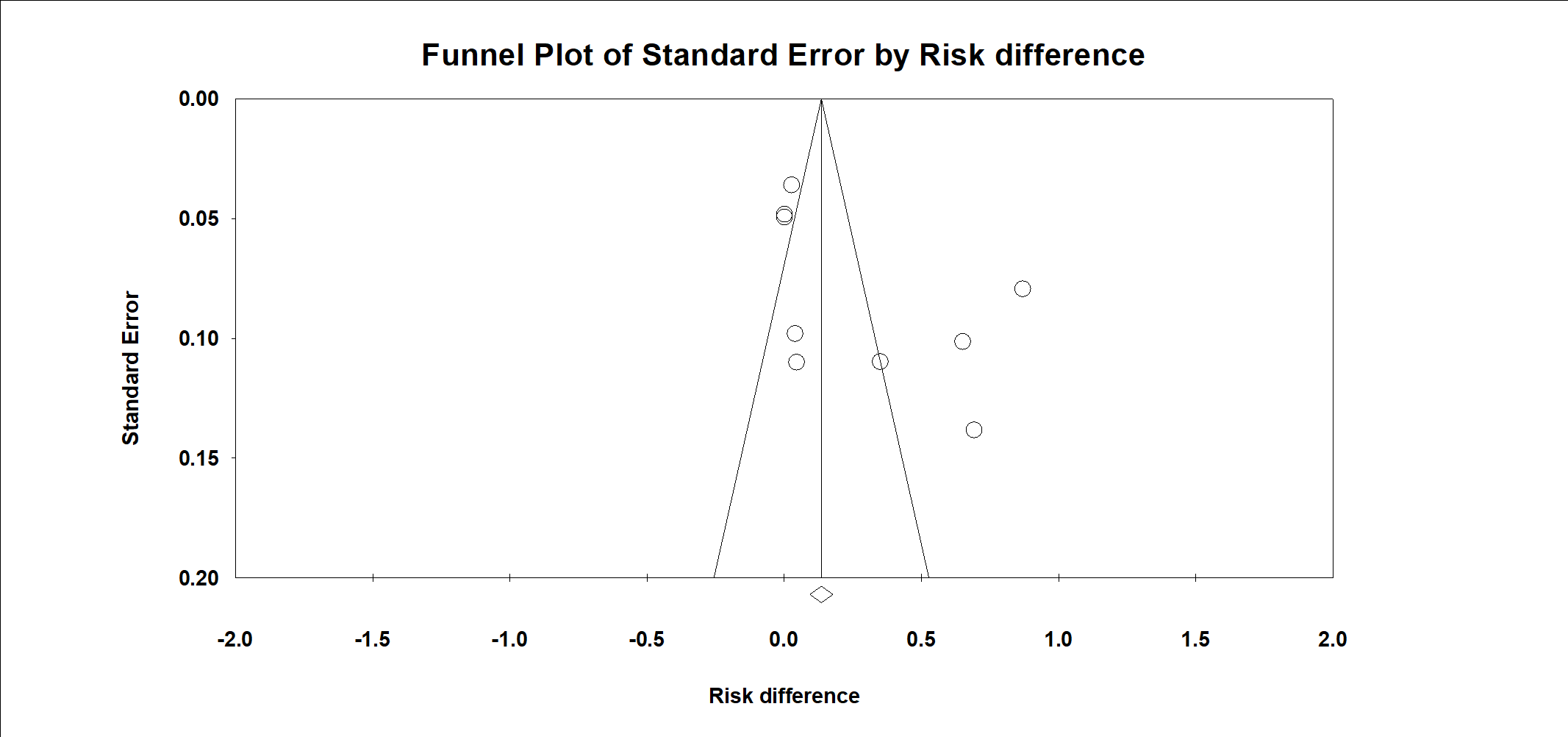

Supplement: S3 Fig — The p value of the Egger’s test was 0.074, indicating probably no evidence of publication bias. (TIF) [file pone.0323051.s003.tif]

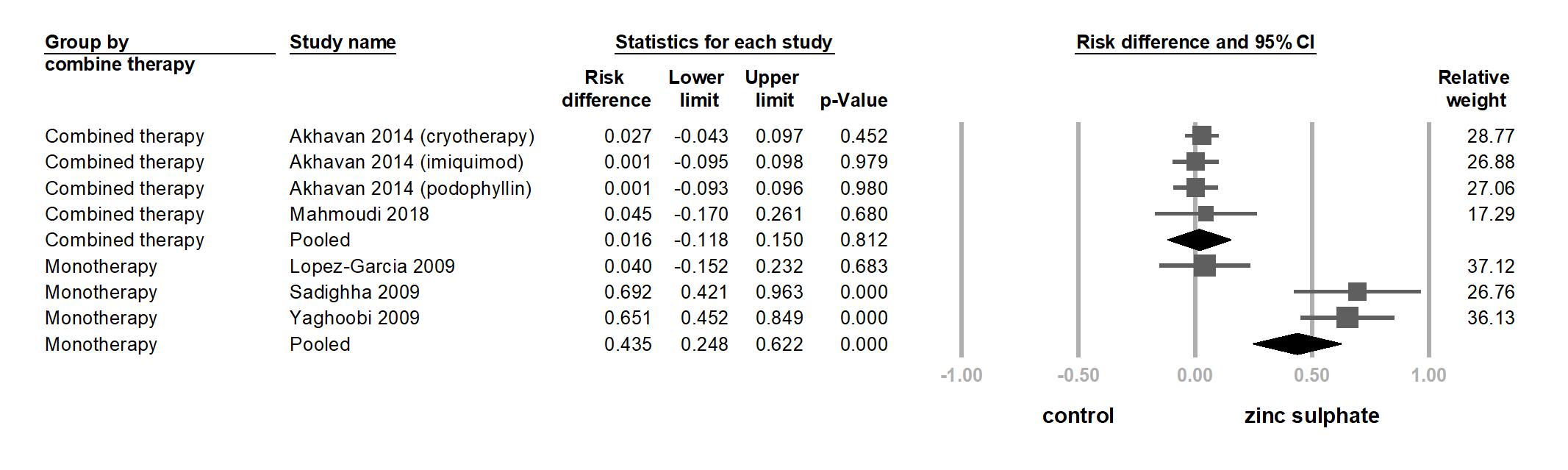

Supplement: S4 Fig — After reanalysis, the results were consistent with the original subgroup analysis. (TIF) [file pone.0323051.s004.tif]
