# Supplementary material for: Oral zinc sulphate reduces the recurrence rate and provides significant therapeutic effects for viral warts: A systematic review and meta-analysis of randomized controlled trials
Source: PLoS One. 2025 May 7;20(5):e0323051. doi: 10.1371/journal.pone.0323051 (PMC12058182; doi:10.1371/journal.pone.0323051)
Supplement: S2 Table — (DOCX) [file pone.0323051.s006.docx]

**Table S2.** Keywords and search results in different databases

| Database | Keyword | Filter | Date | Results |
| --- | --- | --- | --- | --- |
| PubMed | (“wart*” OR “ viral wart*” OR “verruca vulgaris” OR “condyloma acuminatum” OR “verruca plana”) AND (“zinc” OR “zinc sulphate” OR “zinc gluconate”) | NA | July 1, 2024 | 156 |
| Embase | (“wart*” OR “ viral wart*” OR “verruca vulgaris” OR “condyloma acuminatum” OR “verruca plana”) AND (“zinc” OR “zinc sulphate” OR “zinc gluconate”) | Title Abstract  Keyword | July 1, 2024 | 566 |
| Cochrane CENTRAL | (“wart*” OR “ viral wart*” OR “verruca vulgaris” OR “condyloma acuminatum” OR “verruca plana”) AND (“zinc” OR “zinc sulphate” OR “zinc gluconate”) | Title Abstract  Keyword | July 1, 2024 | 48 |
| Web of science | (“wart*” OR “ viral wart*” OR “verruca vulgaris” OR “condyloma acuminatum” OR “verruca plana”) AND (“zinc” OR “zinc sulphate” OR “zinc gluconate”) | NA | July 1, 2024 | 177 |
| ClinicalTrials.gov | (“wart*” OR “ viral wart*” OR “verruca vulgaris” OR “condyloma acuminatum” OR “verruca plana”) AND (“zinc” OR “zinc sulphate” OR “zinc gluconate”) | Condition  or disease | July 1, 2024 | 4 |

NA = not applied.
