# Supplementary material for: Oral zinc sulphate reduces the recurrence rate and provides significant therapeutic effects for viral warts: A systematic review and meta-analysis of randomized controlled trials
Source: PLoS One. 2025 May 7;20(5):e0323051. doi: 10.1371/journal.pone.0323051 (PMC12058182; doi:10.1371/journal.pone.0323051)
Supplement: S4 Table — (DOCX) [file pone.0323051.s008.docx]

**Table S4-1.** All data extracted from the primary research sources regarding the primary outcome.

| Study name | Zinc sulphate group | | Placebo group | | Mean of the initial zinc ion level (μg/dL) | Mean difference of the increase in zinc ion level after treatment (μg/dL) | Mean of the disease duration (month) |
| --- | --- | --- | --- | --- | --- | --- | --- |
|  | Number of total clearances | Total number | Number of total clearances | Total number |  |  |  |
| Gurairi 2002 | 20 | 23 | 0 | 20 | 62.50 | 125.13 | 24.00 |
| Lopez-Garcia 2009 | 4 | 25 | 3 | 25 | 102.00 | 49.00 | N/A |
| Sadighha 2009 | 10 | 13 | 1 | 13 | 53.30 | 107.00 | 27.30 |
| Yaghoobi 2009 | 25 | 32 | 3 | 23 | 55.09 | 103.81 | 28.40 |
| Akhavan 2014 (cryotherapy) | 40 | 40 | 36 | 37 | N/A | N/A | N/A |
| Akhavan 2014 (imiquimod) | 38 | 40 | 37 | 39 | N/A | N/A | N/A |
| Akhavan 2014 (podophyllin) | 39 | 41 | 38 | 40 | N/A | N/A | N/A |
| Moniem 2016 | 7 | 20 | 0 | 20 | 85.95 | 29.66 | 9.25 |
| Mahmoudi 2018 | 26 | 38 | 23 | 36 | 113.77 | 15.46 | 21.90 |

N/A = not applicable, Since the Akhavan 2014 study did not include zinc ion concentration or disease duration in its results, we excluded it from the subgroup analysis and meta-regression.

**Table S4-2.** All data extracted from the primary research sources regarding the relapse rates.

| Study name | Zinc sulphate group | | Placebo group | |
| --- | --- | --- | --- | --- |
|  | Number of relapses | Total number | Number of relapses | Total number |
| Akhavan 2014 (cryotherapy) | 3 | 40 | 11 | 36 |
| Akhavan 2014 (imiquimod) | 1 | 38 | 10 | 37 |
| Akhavan 2014 (podophyllin) | 5 | 39 | 9 | 38 |
| Mahmoudi 2018 | 3 | 26 | 6 | 23 |

**Table S4-3.** All data extracted from the primary research sources regarding the adverse events.

| Study name | Zinc sulphate group | | Placebo group | | Zinc sulphate group | | Placebo group | |
| --- | --- | --- | --- | --- | --- | --- | --- | --- |
|  | Patients who experienced nausea | Total number | Patients who experienced nausea | Total number | Patients who experienced vomiting | Total number | Patients who experienced vomiting | Total number |
| Gurairi 2002 | 23 | 23 | 0 | 20 | 5 | 23 | 0 | 20 |
| Lopez-Garcia 2009 | 10 | 25 | 10 | 25 | 8 | 25 | 0 | 25 |
| Mahmoudi 2018 | 31 | 45 | 2 | 38 | 8 | 45 | 2 | 38 |

Data extractor: Chen-chi, Wang

Date of data extraction: July 13, 2024

Studies listed above are eligible to be included in the review.
