# Supplementary material for: Oral zinc sulphate reduces the recurrence rate and provides significant therapeutic effects for viral warts: A systematic review and meta-analysis of randomized controlled trials
Source: PLoS One. 2025 May 7;20(5):e0323051. doi: 10.1371/journal.pone.0323051 (PMC12058182; doi:10.1371/journal.pone.0323051)
Supplement: Attachment — Submitted filename: Response to Reviewers.docx [file pone.0323051.s011.docx]

**Reviewer #1**

**Q1: Thank you for inviting me to review the paper on the effect of oral zinc sulfate on viral warts. My main concern is why the authors pooled the Akhavan study, which has a different drug dosage, with other studies.**

A1: Thank you very much for taking the time to read my research. It is truly an honor to receive your valuable feedback. In the Akhavan study, the zinc sulfate dosage was fixed at 400 mg/day, which indeed differs from other studies (10 mg/kg/day, with a maximum of 600 mg/day). Currently, there is no literature specifying the exact zinc sulfate dosage for treating warts. However, when referencing studies on other diseases, such as Sharquie's publication (1) on “oral zinc sulfate treatment for leishmaniasis,” it was mentioned that there was no significant difference in treatment efficacy between the 5 mg/kg and 10 mg/kg groups. For an adult weighing 60 kg, the daily intake would be between 300 and 600 mg. Therefore, we believe that keeping the zinc sulfate dosage within this range should be considered a reasonable experimental design, and this is the reason we pooled Akhavan study together with others. We sincerely appreciate your insights.

**Q2: Title: Reduces "the" recurrence rate...**

A2: Thank you for pointing out this significant grammatical error. It has been corrected!

**Q3: The abstract needs to be structured. The search date and the databases searched should be mentioned (method). The number of included studies and the number of studies in your primary search should be mentioned (result).**

A3: Thank you very much for your valuable feedback. We have incorporated the essential elements you mentioned into the abstract. Below is the revised paragraph. [On July 1, 2024, we performed an extensive database search on PubMed, Embase, Web of Science, Cochrane CENTRAL, and ClinicalTrials.gov. Initially, 952 studies were identified, and after screening, 7 studies were included in the final analysis.]

**Q4: Did the authors use MeSH words and other synonyms? Was any language restriction applied?**

A4: Thank you for raising this important question. Although we did not use MeSH terms in our search, we made every effort to include relevant keywords and their synonyms in the search strategy. For example, we accounted for the various terminologies used for warts in different forms and considered the potential formulations of zinc-containing medications. Additionally, we did not impose any language restrictions during the literature search.

**Q5: Summarize the number of studies at each step of the selection of eligible studies in the study selection part. Add the list of excluded studies at the full-text review step and the reasons for exclusion in the supplementary materials.**

A5: Thank you for your feedback. We have updated the content in the manuscript accordingly! The following is the revised section. [Initially, we identified 952 studies. We then removed duplicates (n = 298), studies on unrelated topics (n = 554), and non-RCT studies (n = 60), leaving 40 studies. Next, we excluded studies without full-text availability (n = 2), those not administering zinc ions orally (n = 26), and studies with inappropriate experimental or control group designs (n = 5). This resulted in a final selection of 7 studies for analysis. The literature searching process is presented in “Fig 1”, and all non-duplicate studies (n = 654) identified during the search, along with the reasons for their exclusion, are documented in “S3_Table”.]

**Q6: A funnel plot should not be used for studies with fewer than 10 inclusions.**

A6: Indeed, you are absolutely correct. Conducting a funnel plot with fewer than 10 studies may lead to misinterpretations regarding potential asymmetry. Our main reason for producing the funnel plot is to transparently present the data characteristics, allowing readers to intuitively understand the relationship between the distribution of studies and research findings. Although we have fewer than 10 studies, we also applied Egger's test to check for asymmetry, and the results did not indicate any. Therefore, we interpret the broader horizontal scatter as a reflection of heterogeneity among studies. A possible improvement would be to clearly mention in the Results section that this funnel plot has limitations due to the small number of studies analyzed, which should be interpreted cautiously. [We created a funnel plot for the seven studies that visually showed slight asymmetry. However, Egger's test yielded a two-tailed p value of 0.074, indicating a low likelihood of publication bias. Nonetheless, given the inclusion of fewer than 10 studies, the symmetry should be interpreted with caution.]

**Q7: A table should be added summarizing the results of the statistical analyses of the included studies, such as RR, RD, etc.**

A7: Thank you very much for this valuable feedback; it will make our research findings more accessible to readers. We have now added table 3 and 4 into the paper.

**Q8: Data regarding double/single blinding of the studies should be added to Table 1. The inclusion criteria of the RCTs should be added to the table.**

A8: Thank you very much for this important feedback; it has made the study characteristics more comprehensive. The updated table has been included in the manuscript.

**Q9: Why were these studies only conducted in the Middle East and North Africa region?**

A9: Indeed, you point out an important issue. The majority of the studies included in this paper are from the Middle East and North Africa. We speculate that this may be related to healthcare accessibility in these regions, which might make viral warts a significant concern in these areas. On the other hand, from the data we reviewed, although the prevalence rate of warts among Whites is reported to be twice as high as that among Blacks and Asians, there are also isolated reports indicating a wart prevalence of 3.3% in the United States and as high as 33% in the Netherlands (2, 3). Clearly, there is a notable discrepancy between regional and overall prevalence rates. As there are currently no prevalence reports from the Middle East, we hypothesize that the prevalence of warts may also be relatively high in the Middle East and North Africa, making this a frequent concern for dermatologists in these areas, which may explain the higher number of studies on this topic originating from these regions.

**Reviewer #2**

**Q1: Strengths: This systematic review and meta-analysis offers robust and comprehensive insights into the therapeutic effects of oral zinc sulphate for viral warts. By conducting subgroup analyses based on plasma zinc levels and treatment modalities, the study provides more detailed insights into the efficacy of zinc sulphate under various conditions. The use of statistical methods such as random-effects models, sensitivity analyses, and Egger’s test for publication bias ensures that the conclusions are not only statistically significant but also reliable. Clinically relevant information is particularly highlighted for patients with low plasma zinc levels or those resistant to conventional treatments.**

A1: Thank you very much for taking the time to carefully read our article. We greatly appreciate your detailed insights and thorough understanding of the important aspects of this study. The results presented in this article focus on the effectiveness of oral zinc sulfate in treating viral warts. In addition to reporting the overall outcomes, our study distinguishes itself from other systematic reviews by utilizing subgroup analysis and meta-regression to identify potential factors that may influence treatment efficacy. These factors are then integrated with clinical practice to facilitate shared decision-making between physicians and patients who may consider this therapy.

**Q2: Limitations: Some studies included in the meta-analysis had small sample sizes, which may reduce the statistical power of the conclusions. Additionally, the study population was predominantly female (72.6%), and the impact of zinc supplementation may differ across sexes.**

A2: Indeed, you are correct. One of the weaknesses of this study lies in the smaller sample sizes of certain included studies, as smaller studies may be more prone to showing significant effects. However, we addressed this issue by conducting a sensitivity test, which helps us understand whether the primary outcome remains significant after the sequential removal of individual studies. Regarding the gender distribution, the included studies tended to have a higher proportion of female participants. Unfortunately, we were unable to obtain data on treatment outcomes stratified by gender from the included studies. As a result, we could not perform an additional meta-analysis to evaluate whether the effectiveness of oral zinc sulfate differs between genders. Thank you for your valuable feedback.

**Reviewer #3**

**Q1: It would be better if you could provide evidence for the statement "However, the conclusions of these studies are inconsistent, and comprehensive research has been lacking in recent years."**

A1: Yes, your question is very important, and it has also made us realize that our description of the research background in the article was not sufficiently precise. To strengthen the discussion on the inconsistent evidence in existing literature, here is the revised paragraph. [Existing literature on oral zinc therapy for viral warts shows mixed findings: one clinical trial reported excellent therapeutic effects, with 86% of patients taking zinc supplements achieving complete wart clearance, while none were cured in the placebo group (4). Other studies suggest mild to moderate benefits (5, 6), while some oppose these findings, stating that zinc supplements neither cure viral warts nor provide additional benefits (7-9). Since the conclusions of these studies are inconsistent and comprehensive research has been lacking…]

**Reviewer #4**

**Q1: English writing is generally not indented at the beginning of the line.**

A1: Understood, I have removed the indentation at the beginning of the lines. Thank you for your guidance!

**Q2: Please add to the preamble the inconsistent specifics of the oral zinc findings and add references, which is what is needed to do this study.**

A2: Indeed, we did not provide a detailed description of our study background and the inconsistent outcomes in the existing literature. Allow me to elaborate; here is the revised paragraph. [Existing literature on oral zinc therapy for viral warts shows mixed findings: one clinical trial reported excellent therapeutic effects, with 86% of patients taking zinc supplements achieving complete wart clearance, while none were cured in the placebo group (4). Other studies suggest mild to moderate benefits (5, 6), while some oppose these findings, stating that zinc supplements neither cure viral warts nor provide additional benefits (7-9). Since the conclusions of these studies are inconsistent and comprehensive research has been lacking…]

**Q3: There are ethnographic differences in the incidence of warts, and although the literature is limited, the reason why this was not subgrouped needs to be stated in the limitations.**

A3: Indeed, incorporating ethnicity as a subgroup in our analysis to explore differences in treatment efficacy is a feasible approach. However, the number of studies available on this topic is relatively limited. This limitation would result in certain ethnic subgroups containing only a single study, which significantly reduces the power of these subgroups. We have added these reasons to the limitations section. Below is the revised paragraph. [Secondly, most of the studies included in this meta-analysis originate from the Middle East. Therefore, further research is needed to determine whether this treatment is effective for patients in other regions. While conducting subgroup analyses by ethnicity could be a viable approach to explore differences in treatment efficacy, in this study, the limited number of available articles led to a reduction in power for certain subgroups. As a result, we did not include ethnicity in our subgroup analysis.]

**Q4: Please provide a specific search strategy for each database**

A4: We sincerely apologize for not including the document initially. We have now added the searching strategy used for each database to S2 Table.

**Q5: the search strategy mentions references to included literature as sources for inclusion, this needs to be reflected in the flowchart**

A5: Thank you for your attention to detail; we truly appreciate the time you've taken to review our figures. We acknowledge that we initially overlooked this detail, which was indeed an oversight on our part. We have now added the cross-reference item to Fig 1.

**Q6: Please add the specific number of literature screening entries in the text.**

A6: Thank you for pointing this out; we appreciate your attention to detail, as this was indeed an oversight on our part. Below is the revised paragraph. [Initially, we identified 952 studies. We then removed duplicates (n = 298), studies on unrelated topics (n = 554), and non-RCT studies (n = 60), leaving 40 studies. Next, we excluded studies without full-text availability (n = 2), those not administering zinc ions orally (n = 26), and studies with inappropriate experimental or control group designs (n = 5). This resulted in a final selection of 7 studies for analysis.]

**Q7: The small amount of literature included is still a major concern, especially when the amount of literature is small for regression.**

A7: Indeed, as noted in the first point of the limitations section, this meta-analysis has limitations in terms of the number of studies and the sample sizes of patients included in each study. I fully agree with your insight on this matter. We have made every effort to search for all potentially relevant literature, and we hope that future RCTs from various regions will further investigate this topic. You also mentioned the issue of regression analysis. It is true that conducting meta-regression with a limited number of studies presents challenges and risks; however, we have provided p-values for each meta-regression performed. While the statistical power may not be strong, we believe these findings still hold some reference value.

**Q8: Two systematic evaluations have been conducted, and the strengths of this study are not fully described in the discussion.**

A8: Indeed, you've clearly identified a significant weakness in the article. To clarify the differences between this paper and other existing studies for our readers, we have revised the discussion section. Here is the modified paragraph. [Additionally, the two recently published systematic reviews focused primarily on describing the outcomes and limitations of each clinical trial. While we agree with their summaries regarding the efficacy of oral zinc sulfate, they provided limited analysis of the factors influencing treatment outcomes. This is where our paper adds significant value, as these insights can enhance our understanding of patient selection and the prediction of treatment efficacy.]

**Q9: Why were subgroup analyses of gender not conducted, and if the gender differences were so large, was it possible to extract data for secondary analysis?**

A9: Indeed, your point is very precise. In our included population, the proportion of male participants was significantly lower than female participants. Using gender as a basis for subgroup analysis might reveal differences in the effects of zinc sulphate across genders. However, while all studies provided the number of participants experiencing relief in both the intervention and control groups, they did not specify these figures for males and females separately. This lack of data prevented us from conducting a subgroup analysis by gender. We have addressed this issue in the third part of the limitations section and hope that future studies will consider documenting treatment effects by gender.

**Reviewer #5**

**Q1: Through a systematic review and meta-analysis, this study has reported an evidence on the efficacy of oral zinc sulphate, whether monotherapy or as a combined with traditional therapies compared to placebo or other oral therapies for the treatment of viral warts.**

**Recommendation for improvement:**

**•The study provided new insights, however there are several weaknesses still need to be considered.**

**•Deciding to conduct a systematic review, meta-analysis, and meta-regression to produce new, more accurate and valid evidence, however, the interpretations of the findings are not clearly presented. Therefore, the authors are asked to discuss with biostatistician or epidemiologist to enriched the presentation of findings.**

**•The writing style needs to be considered to make it easier for readers to understand and to implement the findings of this study.**

A1: Thank you very much for patiently reading through our work. The key points you've highlighted are very clear. Our main focus is on analyzing the effectiveness of oral zinc sulfate compared to a placebo, or oral zinc sulfate combined with conventional treatment compared to conventional treatment alone, in treating viral warts. We will respond to each of your questions individually. Regarding the presentation of the research data, we have discussed with experts and made improvements to the content of the subgroup analysis. We used treatment type as the grouping criterion. However, putting “combined therapy” and “monotherapy” in the same figure could mislead readers to think that monotherapy is more effective than combined therapy. We therefore decided to enhance the discussion section to clarify this figure. Additionally, two studies used no-treatment and glucose controls instead of starch. After consulting with statisticians, we decided to include a supplementary figure excluding these studies, which still supports our conclusions. Through these adjustments, we hope to clearly communicate our study's findings and data interpretation to readers. I apologize if my English writing may be somewhat difficult to read. To make my research findings clearer, I have added a table summarizing all analysis conclusions, hoping this will help readers more easily grasp the overall results of the study.

**Q2: The background that has become the basis for this research is not strong enough to describe the existing problems. What are the research questions that the authors want to answer? Are all parts (population, intervention, comparison, outcome) that have become the target of the authors supported by data, for example from results of previous research or from existing theories are to be tested? The authors are asked to consider on how is this background related to the research conducted that can help strengthen the authors’ hypotheses. Therefore, the authors are advised to explain the relevance of the background presented.**

A2: Indeed, the original content did not clearly address the questions I aimed to answer, and the background was insufficiently described. Thank you for this opportunity to refine my argument. Below is the revised paragraph. [Many treatments for warts are available, such as cryotherapy commonly used for nongenital warts, topical podophyllin and imiquimod for external anogenital warts, antiviral medications, and immunotherapies such as zinc supplements. However, cryotherapy can cause pain, blistering, and scarring, while topical podophyllin or imiquimod may lead to local skin irritation and are not safe during pregnancy. Therefore, simply taking seemingly harmless zinc supplements to treat viral warts appears to be an attractive option.]

**Q3: In this background section, it would be better to also explain the results of previous primary studies that describe of how effective the zinc sulphate can be as monotherapy or combined therapy, or how the intervention might work compared with others.**

A3: Thank you for your valuable feedback; I fully agree with your perspective. I have therefore incorporated key findings from some of the literature into this paragraph. Below is the revised version. [Existing literature on oral zinc therapy for viral warts shows mixed findings: one clinical trial reported excellent therapeutic effects, with 86% of patients taking zinc supplements achieving complete wart clearance, while none were cured in the placebo group (4). Other studies suggest mild to moderate benefits (5, 6), while some oppose these findings, stating that zinc supplements neither cure viral warts nor provide additional benefits (7-9). Since the conclusions of these studies are inconsistent and comprehensive research has been lacking…]

**Q4: Line 48: The authors stated, “… with the highest prevalence observed in school-aged children and young adults”. Are the results of your study in line with this statement? The results you have reported are the mean (+SD) age of 31.64+12.49. If it is in accordance with the results of previous research that is us as a reference, then it needs to be emphasized in the discussion section.**

A4: Indeed, the prevalence of viral warts is high among children and young adults, which is also supported by the demographic data in this meta-analysis. I will reference this statement from the introduction again in the discussion section. Thank you very much for your attention to detail. [Demographic data showed a mean patient age of 31.64 ± 12.49 (SD), aligning with real-world epidemiological findings indicating the high prevalence among young adults.]

**Q5:“According to the current literature, both topical and oral zinc formulations may be effective as primary or adjunct therapies for viral warts”. However, this systematic review and meta-analysis only analyzed the effectiveness of oral administration. The authors are asked to explain the reason why this study only focused on oral administration.**

A5: Indeed, you are absolutely right; such wording would distract readers from the effectiveness of oral zinc sulfate and doesn’t explain why we chose to analyze oral rather than topical formulations. Allow me to explain. We decided not to include topical formulations for two main reasons. First, unlike oral doses, topical applications cannot be precisely quantified, which may lead to inconsistent dosing and affect the reliability of efficacy results. Second, there was considerable variation in the active ingredients used in clinical trials of topical agents, as well as inconsistencies in the design of control groups. Although these differences could be addressed through subgroup analysis, the overall reliability of the findings remains a concern. Given the potential for this section to confuse readers, and that explaining these choices could obscure the main focus of our analysis, we believe removing it and instead reinforcing the background issues discussed in “Q2” and “Q3” would be a more effective approach.

**Q6: “P, human participants with skin or external genital warts”. The authors are asked to provide scientific reasons why only skin warts and external genital warts are included in the criteria, while all types of warts need to be treated.**

A6: Thank you for raising a valuable point, and I’d be happy to clarify. HPV infections on skin and mucous membranes can be categorized as follows:

1. **Cutaneous wart**: This includes common warts (verruca vulgaris, making up 70% of cutaneous cases), plantar warts (about 30% of the cases), and flat warts (verruca plana). (10)
2. **External genital wart**: Like genital warts, these are sexually transmitted infections. Among them, external genital warts are the most prevalent type. They typically appear on the penis, groin, scrotum, vulva, perineum, and the external or perianal areas. (11)
3. **Genital wart**: They are found in areas such as the urethral meatus, vagina, cervix, or intra-anal regions. (11)
4. **Epidermodysplasia verruciformis**: A rare congenital disease, often linked to autosomal recessive inheritance, with HPV infection. It may also manifest in individuals with acquired immunodeficiencies, such as patients with HIV, organ transplant recipients, or those with lymphoma. (10)

Our analysis focuses on the first two categories for several reasons. First, given that the first two topics represent the most common types of HPV skin and mucosal infections, focusing on this type of infection as the research target seems more aligned with clinical concerns. Second, we found no studies using oral zinc to treat genital warts (those located in urethral meatus, vagina, cervix, or intra-anal regions). Third, HPV infections in the cervix not only cause cervical warts but also increase the risk of cervical cancer. Including cancer as an analysis topic could shift the focus of this paper. Finally, cases of epidermodysplasia verruciformis are rare, making it challenging to gather sufficient studies for analysis.

Therefore, we selected cutaneous warts and the more common external genital warts for our study.

**Q7: The authors stated “the exclusion criteria were..1) non-RCTs; 2) RCTs not involving oral zinc sulphate supplementation , 4) non-placebo-controlled,,,” Explanation of the things that were exclusion criteria are actually already described as inclusion criteria. For example, in the inclusion criteria it is clearly written that studies with RCTs design will be included. This means (without needing to be written again) that studies with designs other than RCTs will be excluded, so it may not need to be sharpened again by writing it in the exclusion criteria section.**

A7: You have indeed pointed out a shortcoming in my writing, and I greatly appreciate your constructive feedback. The original exclusion criteria may have been overly detailed and unnecessarily lengthy. I have now removed the redundant criteria, and here is the revised paragraph. [The inclusion criteria for this study were as follows: 1) randomized controlled trials (RCTs) with human participants; 2) RCTs including the number of individuals who experienced viral wart clearance after treatment in both experimental and control groups; 3) placebo-controlled RCTs (without any age or treatment duration restrictions); and 4) RCTs including those administered a dosage of zinc sulphate…… The exclusion criteria were as follows: 1) RCTs not involving oral zinc sulphate supplementation; and 2) overlapping participants.]

**Q8: In the literature search strategy, the authors only limited the search by using 5 electronic databases. The authors should explain this, as well as explain whether they used grey literature or hand-searching to recruit includes study in this systematic review.**

A8: Thank you for raising this question, and I deeply appreciate your dedication to thorough research. Allow me to address your inquiry.

In this meta-analysis, we utilized the databases PubMed, Embase, Web of Science, Cochrane CENTRAL, and ClinicalTrials.gov. According to Section 4.3.1.1, Box 4.3.a of the Cochrane Handbook, the minimum requirement for comprehensive literature searching includes the Cochrane Review Group’s (CRG’s) Specialized Register, MEDLINE, and Embase.

1. The Cochrane Review Group’s Specialized Register can be accessed externally by searching Cochrane CENTRAL.
2. The MEDLINE database can be searched via PubMed, which provides free access to MEDLINE and includes additional journals outside MEDLINE’s scope.
3. Embase not only includes all MEDLINE records but also indexes an additional 3,000 journals and 10 million records.
4. Additionally, a 2015 study found that searching PubMed alone can cover between 75-92% of relevant literature (12), and a 2016 study suggested that using just two databases can capture most studies (13). For example, one study cited that using only CENTRAL and MEDLINE can achieve substantial coverage (14).
5. Based on these recommendations from the Cochrane Handbook, we included PubMed (to access MEDLINE), Cochrane CENTRAL (to access CRG’s Specialized Register), and Embase, as well as Web of Science for a broader search.
6. The Cochrane Handbook requires searching trials registers; therefore, we included ClinicalTrials.gov as a representative source for this category.
7. As for "other resources," while we did not include grey literature or conduct hand-searching, we reviewed other systematic reviews and screened their reference lists for potentially relevant articles to include in our analysis.

This is our reasoning for selecting this number of databases. Although grey literature and hand-searching are not mandatory, we acknowledge that their absence is a limitation of this study. Thank you for your precise and valuable feedback!

**Q9: The keywords compiled by the authors seem to describe only intervention and outcome. Therefore, authors are asked to consider developing a search strategy by enriching the keywords that include participants and comparisons as well. Boolean technique and the use of more alternative words need to be considered.**

A9: Indeed, our keyword selection primarily focused on the intervention method and its corresponding outcomes, without specifically targeting keywords for participants or comparison groups. Our rationale was that using more restrictive keywords could risk excluding relevant studies. Therefore, we first conducted the search using our current keywords and then performed manual screening and exclusion based on titles and abstracts. For example, if we had included terms for participants based on our PICO framework, we would have added 'Humans' as a Mesh term. In PubMed, our original search returned 158 results, but adding the new keyword [("Humans"[Mesh]) AND ("wart*" OR "viral wart*" OR "verruca vulgaris" OR "condyloma acuminatum" OR "verruca plana") AND ("zinc" OR "zinc sulphate" OR "zinc gluconate") AND ("clearance" OR "cure" OR "response" OR "relapse" OR "recurrence" OR "adverse events" OR "side effects")] resulted in only 33 results. Additionally, we included synonyms for warts in various locations and forms to ensure comprehensive coverage of the literature.

**Q10: The authors are asked to explain if there is any language restriction, and the reasons to limit it.**

A10: Thank you for your question. We didn’t apply any language restrictions in our search; this clarification has now been added to the manuscript. [The literature search included articles from the earliest available records up to July 1, 2024, without year and language restrictions.]

**Q11: “If the two authors had different opinions during the process, a third author was consulted to resolve any disagreements”. There, the authors are asked to explain if there is any disagreement, what are them, and how to solve them.**

A11: Thank you for your attention to detail. In fact, throughout the process of conducting our literature search and developing our methodology, we found it easy to reach consensus as our research questions and intervention methods were clearly defined. At no point did we encounter disagreements that required a third author to make the final decision.

**Q12: The secondary outcome was defined as the rate of relapse occurring within six months after treatment as well as any treatment-related side effects. Please be explained clearly if you analyses only for studies reported rate of relapse at least 6 months after intervention.**

A12: Thank you for reading carefully and for your question; you've indeed identified a writing oversight on our part. In this meta-analysis, the secondary outcomes were set as relapse rate and treatment-related adverse events. For the relapse rate, we did not specify that data had to be analyzed based on a certain number of follow-up months, as we stated in the synthesis method (“Secondary outcomes, including relapse and treatment-related side effects...”) and in the results section (“We analyzed the studies that provided data on the number of relapsed cases during the follow-up period”). We simply selected studies that provided relapse data for analysis, and it happened that all of these studies had a 6-month follow-up period. This led us to interpret in the discussion that “oral zinc sulfate can reduce the 6-month relapse rate.” Therefore, the description in the data collection process section was indeed an inadvertent error, as we did not limit our analysis to the 6-month relapse rate alone. We apologize for this and have made the necessary correction. [The secondary outcome was defined as the rate of relapse after treatment as well as any treatment-related side effects.]

**Q13: To consistent with the results especially for randomization (in line 185: “…three studies were classified as having some risk of bias in the randomization process because they did not describe the concealment of random allocation”), the authors are suggested to define clearly about the randomization.**

A13: Indeed, we did not elaborate on the standards for reviewing randomization in the Methods section, which may lead to reader confusion. Allow me to provide a complete explanation, as follows. [Regarding the standards for reviewing randomization, the allocation sequence must first be kept concealed; for example, the allocation process should be conducted by an external institution or organization. Additionally, the allocation sequence should be unpredictable, with the article mentioning methods such as simple randomization, blocked randomization, or any elements involving random assignment (e.g., computer-generated random numbers or dice rolling). Finally, baseline imbalances need to be assessed.]

**Q14: “Articles without full-text availability were excluded”. Please be explained if any effort in finding out this article, or how the authors solve this issue.**

A14: Thank you for your thorough question; allow me to explain. During our data screening, we identified two studies that, while relevant to the topic, were inaccessible in full text. The first is titled “A Randomized Controlled Trial to Examine the Effectiveness of 400 mg of Oral Zinc Gluconate as Adjunctive Therapy for Anogenital Warts,” registered on ClinicalTrials.gov. This trial is currently listed as “Terminated,” and no data has been uploaded to the website. The second study, “Oral Zinc Gluconate as Treatment for Recalcitrant Cutaneous Warts: A Randomized, Double-blind, Placebo-controlled Trial,” is also registered on ClinicalTrials.gov. Although the trial was completed in September 2013, the study results were not uploaded. We searched for the full text through PubMed and Embase, and the sponsoring organization, the Philippine Dermatological Society, and the implementing hospital, the Philippine General Hospital Section of Dermatology, but could not locate the full text. For these reasons, these two studies were excluded.

**Q15: “… and we ensured that all zinc sulphate studies were administered orally.”. This statement should be in line with inclusion criteria**

A15: Indeed, this text would be more appropriately placed in the inclusion criteria; We have updated the inclusion criteria, and below is the revised paragraph. [The inclusion criteria for this study were as follows……; and 5) studies on the oral application of zinc ions.]

**Q16: “27.4% of whom were male”. In this part, why are male highlighted, usually what is highlighted is the dominant characteristic, in this case are women. Unless there is a specific reason of male participation.**

A16: Thank you very much for taking the time to read my article carefully, and I appreciate you noticing this detail. We initially highlighted the male gender as a representative proportion simply because the number of male participants was relatively low in the included studies, which seemed noteworthy. However, in standard writing practice, I agree that using the dominant characteristic (in this case, female) as the basis would be more appropriate. Below is the revised paragraph. [The seven included RCTs comprised 594 participants, 73.6% of whom were female.]

**Q17: “Based on the available data, the average age was 31.64+12.49” It would be better if these findings are explained briefly in the discussion section, if necessary, because in the introduction section, the authors pay attention to the age variable so that it will be seen whether your results support the explanation in the introduction.**

A17: We believe this approach creates a good correspondence between the introduction and discussion sections, so we have revised the original paragraph accordingly. Thank you very much! [Demographic data showed a mean patient age of 31.64 ± 12.49 (SD), aligning with real-world epidemiological findings indicating the highest prevalence among young adults.]

**Q18: Table 1: The authors stated that data from the *Akhavan, 2014* study (either Cryotherapy or Imiquimod) the comparator was a cream, not an oral preparation. It would be better if this be explained in the methods or inclusion criteria that the comparator is not limited to oral preparations only.**

A18: Yes, I believe your consideration is thorough, and this will help reduce any potential confusion for readers of my article. I will incorporate this section into the PICO framework. [The population (P), intervention (I), comparison (C), and outcome (O) for this study…… C, either taking a placebo or using traditional treatments, e.g., topical drugs or cryotherapy, not limited to oral medications, for viral wart]

**Q19: “Unexpectedly, longer disease duration was associated with better treatment outcomes”. The authors are mentioned this issue in the Discussion as disagreement with only one RCT. Therefore, the authors are asked to suggest in Conclusion section what kind of research could be done to strengthen this finding.**

A19: Indeed, this article should adopt a cautious approach in interpreting the relationship between disease duration and treatment efficacy. This aspect should also be included as an evaluation criterion in future RCTs and meta-analyses studying this therapy. Below is the revised paragraph. [Future research should focus on the safety of zinc sulphate at higher concentrations, considering cases from other regions or ethnic groups, and explore the relationship between the disease duration and its impact on the treatment efficacy for viral warts.]

**Q20: Figure 2: Process of randomization was less than 60%, and overall low risk of bias of included studies was less than 50%. The authors are asked to explain about the risk of bias of included studies, in line with decided to do meta-regression. That may affect the methodological quality of the study.**

A20: Indeed, your insights are very precise; please allow me to explain. I will focus on explaining the review process for the articles rated as having “some risks.”

1. For the randomization process, articles rated as having “some risks” only mentioned that they were randomized controlled trials without specifying the detail of the allocation sequence they applied. Consequently, we could not confirm if the allocation sequence was fully concealed, so we rated this aspect (Question 1.2) as “No information.” Next, all three articles mentioned that no significant differences in gender, age, or disease duration were found between the intervention and control groups. Therefore, we believe there is no baseline imbalance issue, and rated this question (Question 1.3) as “No.” These three articles were ultimately categorized under the randomization process as having “some risks.”
2. In outcome measurement, two articles rated as “some risks” used appropriate methods to measure outcomes (Question 4.1) and applied the same evaluation method to both intervention and control groups (Question 4.2). However, since neither study described blinding, we rated the item on whether the observers knew if patients received the intervention (Question 4.3) as “Probably yes.” In this case, observers may have been influenced by their knowledge of the intervention, potentially impacting their assessment of efficacy (Question 4.4), so this item was rated as “Probably yes.” However, there was no evidence in either article suggesting a strong preconceived belief in the intervention's efficacy; rather, they objectively described their observations. We therefore believe that the authors' knowledge of the treatment likely did not affect the observed outcomes (Question 4.5), rating it as “Probably no.” Consequently, these two articles received an “some risks” rating for outcome measurement.
3. You may have noted that *Gurairi 2002* was a single-blinded study, where the observers were aware of the intervention, so we rated Question 4.3 as “Yes.” However, this article was the first RCT on the use of oral zinc sulfate for viral warts and the first to examine the relationship between zinc ion levels and viral warts. There was no indication of strong pre-existing evidence supporting the therapy’s efficacy for viral warts before the study, so we believe that the observers' assessments may not have been influenced by their knowledge of the intervention. Thus, we rated Question 4.4 as “Probably no,” resulting in an overall “low risk” rating for this study.

**Q21: Figure 6: the authors are asked to explain on the reasons to combine the combined therapy and monotherapy in one.**

A21: Thank you for your question; please allow me to clarify. As with other subgroup analyses, we included all studies directly and then grouped them, pooling the data within each subgroup. This is why both groups' data appear on the same chart. Displaying them on the same chart allows us to more intuitively examine whether their confidence intervals overlap. However, as mentioned in our response to the first question, comparing these two treatment approaches side by side may lead readers to mistakenly believe that monotherapy is superior to combined therapy. This misconception is something we have further clarified in the discussion section.

**Q22: Supplement figure 4: the authors are asked to explain the results, where 5 out of 8 studies are outside the line.**

A22: Thank you for your question; I will do my best to explain. Although we included fewer than 10 studies, which may limit the power of a funnel plot, we still conducted an Egger's test, which showed a non-significant result, indicating symmetry in the plot. Additionally, this meta-analysis used a random effects model, which is often used to incorporate heterogeneity in meta-analyses. If the heterogeneity fits with the assumptions of this model, a funnel plot will be symmetrical but with additional horizontal scatter (15). Additionally, among studies of similar size, the three (Gurairi 2002, Sadighha 2009, and Yaghoobi 2009) on the right side of the plot appear to have larger effect sizes, which may also relate to the study design and the quality of those trials. Considering these points, we believe that some studies falling outside the lines (which represent the 95% confidence range) may result from heterogeneity and differences in individual study designs and quality.

**Q23: The high heterogeneity may also be caused by inconsistent of operational definition of variables, design, or dosage of zinc sulphate, and duration for therapies.**

A23: You're absolutely correct; there is substantial heterogeneity among the studies included in this meta-analysis. This is partly due to the limited number of studies available on this topic and the small sample sizes in some of them. However, we have attempted to explore the underlying reasons affecting efficacy by conducting subgroup analyses and meta-regression to address the heterogeneity issue. Additionally, most studies did not specify the exact doses of zinc sulfate administered, only mentioning weight-based dosing without providing weight distributions. This limits our ability to examine the influence of dosage on efficacy. These factors indeed represent limitations of this paper.

**Q24: The Discussion section are provided insight findings. However, the authors are lacking in explanation of the agreement and disagreement of this findings with previous studies or literature theories. The authors are asked to clearly distinguish finding gap in the background and how your results contribute to the field.**

A24: Indeed, you have clearly identified a key weakness in our article. We did not explicitly indicate which perspectives from previous studies we agree with, nor did we highlight the potential contributions of this paper to the dermatology field. After re-evaluating the prior literature, we revised certain paragraphs accordingly. [Additionally, the two recently published systematic reviews focused primarily on describing the outcomes and limitations of each clinical trial. While we agree with their summaries regarding the efficacy of oral zinc sulfate, they provided limited analysis of the factors influencing treatment outcomes. This is where our paper adds significant value, as these insights can enhance our understanding of patient selection and the prediction of treatment efficacy.]

**Q25: The authors are suggested to enriched their arguments, incorporate multiple viewpoints from existing literature to provide a balanced understanding of the topic.**

A25: We completely agree with your opinion. We believe it is important to incorporate perspectives from different angles in the article to achieve an objective and balanced commentary on the subject. Below is the paragraph in which we applied this concept while writing the article.

1. **Perspectives on the clinical efficacy of zinc ions**: [A previous prospective study comparing the efficacy of oral zinc sulphate and valacyclovir for wart treatment found that valacyclovir was superior. Another open-label study reported a cure rate of 50% in patients treated with oral zinc sulphate. Two systematic review summarized various zinc-related therapies for wart treatment and highlighted the efficacy of oral zinc sulphate], [studies have shown that warts are more refractory to treatment and are more likely to recur in patients with zinc deficiency].
2. **Discussion on the possible mechanisms of action for the efficacy of zinc ions**: [Some studies suggest that zinc can regulate the translation of interferon-γ……Other studies have suggested that zinc promotes and inhibits the signaling pathways of interferon receptors through phosphorylation and dephosphorylation……], [Exogenous zinc supplementation can effectively inhibit the synthesis of E6 and E7 proteins, leading to apoptosis of dysplastic cervical cancer cells……zinc can inhibit the enzymatic activity of viral proteases and polymerases and affect the processes of attachment, infection, and uncoating].
3. **Explanation of the mechanisms behind low zinc ion efficacy and an exploration of the underlying sociogeographical factors:** [……in rural areas where diets often include a high intake of whole wheat bread. This dietary habit is rich in fiber and phytates, which can reduce zinc absorption efficiency, potentially leading to zinc deficiency]
4. **Different perspectives on zinc ion dosage**: [In fact, for patients with malabsorption syndromes, such as Crohn’s disease or short bowel syndrome, the dose for treating acute zinc deficiency is typically only 50 mg/d. Exceeding this dose can lead to gastrointestinal side effects such as nausea, vomiting, and gastric bleeding. Daily intake of more than 150 mg of zinc can potentially affect lipid profiles and immune function status. Excessive zinc intake may also interfere with copper absorption, leading to copper deficiency]

**Q26: The authors are also advised to discuss deeply about the potential of the limitations of the study in the end of Discussion section.**

A26: You are absolutely correct; the limitations should be presented and explained at the end of the discussion. Below is our specific description of the limitations in this article.

1. **Highlight the limitations of the literature included in this article and the heterogeneity between studies, and propose corresponding approaches such as subgroup analysis and meta-regression**: [First, the number of available studies on this topic was limited……some studies had small sample sizes or high dropout rates, which may have contributed to significant heterogeneity and potential bias in the results. To address this, we performed subgroup analyses based on the “initial plasma zinc ion concentration” and “whether combined therapy was used” to examine factors that could contribute to heterogeneity. We also performed a meta-regression to identify the relationship between treatment efficacy and the increase in zinc ion levels as well as the duration of the disease.]
2. **Highlight the geographic limitations of the included literature and explain the rationale for not using geographic data as a criterion for subgroup analysis:** [Secondly, most of the studies included in this meta-analysis originate from the Middle East. Therefore, further research is needed to determine whether this treatment is effective for patients in other regions. While conducting subgroup analyses by ethnicity could be a viable approach to explore differences in treatment efficacy, in this study, the limited number of available articles led to a reduction in power for certain subgroups. As a result, we did not include ethnicity in our subgroup analysis.]
3. **Highlight the gender ratio differences among patients included in this article and raise the question of whether the drug has the same efficacy across genders, suggesting this as a direction for future research**: [Third, the sex distribution of the patients included in this study was imbalanced, with only 27.4% of the patients being male. However, whether zinc sulphate is particularly effective in female remains unclear. Most of the existing literature has not analyzed sex as a subgroup. Future studies should address this issue.]

**Q27: The authors are suggested to explain any unique contributions, potential applications, or scientific and policy implementation of the findings.**

A27: We describe the applications and potential benefits of zinc sulfate in various clinical contexts, and incorporate the concept of shared decision-making to balance medical evidence with patient preferences. [Oral zinc sulphate monotherapy can provide significant therapeutic effects in patients unwilling to undergo conventional treatment. For patients using traditional treatments for viral warts, the addition of zinc sulphate can significantly reduce the recurrence rate over the next six months.]

Reference

1.	Sharquie KE, Najim RA, Farjou IB, Al-Timimi DJ. Oral zinc sulphate in the treatment of acute cutaneous leishmaniasis. Clin Exp Dermatol. 2001;26(1):21-6.

2.	Van Haalen FM, Bruggink SC, Gussekloo J, Assendelft WJJ, Eekhof JAH. Warts in primary schoolchildren: prevalence and relation with environmental factors. British Journal of Dermatology. 2009;161(1):148-52.

3.	Silverberg JI, Silverberg NB. The US Prevalence of Common Warts in Childhood: A Population-Based Study. Journal of Investigative Dermatology. 2013;133(12):2788-90.

4.	Al-Gurairi FT, Al-Waiz M, Sharquie KE. Oral zinc sulphate in the treatment of recalcitrant viral warts: randomized placebo-controlled clinical trial. Br J Dermatol. 2002;146(3):423-31.

5.	Moniem EA, Genedy RM, Moussa R. Oral zinc sulfate in the treatment of recalcitrant warts. Egyptian Journal of Dermatology and Venerology. 2016;36(2).

6.	Yaghoobi R, Sadighha A, Baktash D. Evaluation of oral zinc sulfate effect on recalcitrant multiple viral warts: A randomized placebo-controlled clinical trial. Journal of the American Academy of Dermatology. 2009;60(4):706-8.

7.	Akhavan S, Mohammadi SR, Modarres Gillani M, Mousavi AS, Shirazi M. Efficacy of combination therapy of oral zinc sulfate with imiquimod, podophyllin or cryotherapy in the treatment of vulvar warts. Journal of Obstetrics and Gynaecology Research. 2014;40(10):2110-3.

8.	López-García DR, Gómez-Flores M, Arce-Mendoza AY, de la Fuente-García A, Ocampo-Candiani J. Oral zinc sulfate for unresponsive cutaneous viral warts: too good to be true? A double-blind, randomized, placebo-controlled trial. Clin Exp Dermatol. 2009;34(8):e984-5.

9.	Mahmoudi H, Ghodsi SZ, Tavakolpour S, Daneshpazhooh M. Cryotherapy plus oral zinc sulfate versus cryotherapy plus placebo to treat common warts: A double blind, randomized, placebo-controlled trial. International Journal of Women's Dermatology. 2018;4(2):87-90.

10.	Saavedra AP, Roh EK, Mikailov A. Viral Diseases of Skin and Mucosa. Fitzpatrick’s Color Atlas and Synopsis of Clinical Dermatology, 9e. New York, NY: McGraw-Hill Education; 2023.

11.	Saavedra AP, Roh EK, Mikailov A. Sexually Transmitted Diseases. Fitzpatrick’s Color Atlas and Synopsis of Clinical Dermatology, 9e. New York, NY: McGraw-Hill Education; 2023.

12.	Halladay CW, Trikalinos TA, Schmid IT, Schmid CH, Dahabreh IJ. Using data sources beyond PubMed has a modest impact on the results of systematic reviews of therapeutic interventions. J Clin Epidemiol. 2015;68(9):1076-84.

13.	Hartling L, Featherstone R, Nuspl M, Shave K, Dryden DM, Vandermeer B. The contribution of databases to the results of systematic reviews: a cross-sectional study. BMC Med Res Methodol. 2016;16(1):127.

14.	Nussbaumer-Streit B, Klerings I, Wagner G, Heise TL, Dobrescu AI, Armijo-Olivo S, et al. Abbreviated literature searches were viable alternatives to comprehensive searches: a meta-epidemiological study. J Clin Epidemiol. 2018;102:1-11.

15.	Sterne JA, Sutton AJ, Ioannidis JP, Terrin N, Jones DR, Lau J, et al. Recommendations for examining and interpreting funnel plot asymmetry in meta-analyses of randomised controlled trials. Bmj. 2011;343.
